# First Report of Pinnatoxin-G (PnTX-G) in a Marine–Coastal Area of the Adriatic Sea Associated with the Presence of the Dinoflagellate *Vulcanodinium rugosum*

**DOI:** 10.3390/md22030122

**Published:** 2024-03-05

**Authors:** Monica Cangini, Sonia Dall’Ara, Silva Rubini, Barbara Bertasi, Paolo Rizzi, Giovanni Dell’Orfano, Stefania Milandri, Stefano Manfredini, Erika Baldini, Silvia Vertuani

**Affiliations:** 1 Fondazione Centro Ricerche Marine, National Reference Laboratory for Marine Biotoxins, Viale A. Vespucci 2, 47042 Cesenatico, Italy; monica.cangini@centroricerchemarine.it (M.C.); sonia.dallara@centroricerchemarine.it (S.D.); stefania.milandri@centroricerchemarine.it (S.M.); 2 Experimental Zooprophylactic Institute of Lombardy and Emilia Romagna, Via Modena 483, 44124 Ferrara, Italy; silva.rubini@izsler.it; 3 Experimental Zooprophylactic Institute of Lombardy and Emilia Romagna, Via A. Bianchi 9, 25124 Brescia, Italy; barbara.bertasi@izsler.it; 4 AUSL Ferrara—Operational Unit “Food Hygiene of Animal Origin”, Via Cassoli 30, 44121 Ferrara, Italy; p.rizzi@ausl.fe.it (P.R.); giovanni.dellorfano@ausl.fe.it (G.D.); 5 Department of Life Sciences and Biotechnology, Faculty of Medicine, Pharmacy and Prevention, University of Ferrara, Via Luigi Borsari 46, 44121 Ferrara, Italy; smanfred@unife.it (S.M.); vrs@unife.it (S.V.)

**Keywords:** emerging toxins, drug discovery, cyclic imines, *Vulcanodinium rugosum*

## Abstract

This study reports the first detection of the marine neurotoxin pinnatoxin-G (PnTX-G) in clams collected in the northwestern Adriatic Sea (Italy). It also represents the first report of the potential toxin-producing dinoflagellate, *Vulcanodinium rugosum*, in Italian seas. This result, from the coasts of the Emilia-Romagna Region, indicates a successful colonization process, reflecting conditions in France where *V. rugosum* was initially documented. In this case, the concentration of PnTXs was very low, making further sampling necessary to fully understand the extent of the phenomenon. Discussions on the need to obtain more data to support a proper risk assessment and the need to implement a monitoring program that includes emerging marine biotoxins are also included.

## 1. Introduction

Pinnatoxins (PnTXs) are swift-acting neurotoxins, paralytic chemical compounds that inhibit neuronal and muscle-type nicotinic acetylcholine receptors. Eight different subtypes, designated pinnatoxin A–H, have been described, being PnTX-G the most commonly detected toxin in mollusks. A summary can be found in Table 1.

Secreted by the dinoflagellate *Vulcanodinium rugosum* (Nézan and Chomérat, 2011) PnTXs have been observed to accumulate in bivalve mollusks since their discovery in 1990. PnTXs were first identified in the bivalve *Pinna attenuata* (Reeve, 1858) collected in 1990 in the Guangdong region, on the southeastern coast of China [1]. Isolates of PnTX-A to PnTX-D were obtained from another species of Pinna, *P. muricata* Linnaeus, in Japan [2,3,4].

The variants PnTX-E to PnTX-G have been located in oysters and various bivalves from New Zealand to Australia [5,6,7]. PnTX-H was traced to the South China Sea from a culture of *V. rugosum* [8]. Within Europe, PnTXs are deemed emerging marine biotoxins, alongside 40 other compounds, including spirolides, palytoxins, ciguatoxins, tetrodotoxins, and brevetoxins, whose activities are not yet fully understood [9,10]. *V. rugosum*, identified in 2011 [11,12,13], is currently the sole confirmed algal producer of PnTXs.

PnTX-G has been reported in various regions including Australia, New Zealand, Japan [12,14], southern Chilean waters [15,16], the Pacific coast of Mexico [17], the South China Sea [7], the Gulf of Thailand [18], the Arabian Gulf [19], and in Canadian mussels in 2012 [20]. The toxins were first noted in Europe in Norwegian mussels in 2009 [21].

In 2011, PnTXs were discovered in mollusks from the Mediterranean coast of France [11], with subsequent identifications in France [9,22,23,24] and Spain [25,26,27]. The first report of PnTXs in Italy was by Varriale et al. in 2021, involving Sardinian mussels [28].

PnTXs are highly toxic to mice via intraperitoneal injection, but their toxicity decreases when administered orally [6,29]; however, PnTXs are unusual in that the difference between their oral and intraperitoneal toxicity is less than other toxins [29]. Moreover, it has been demonstrated in rats that PnTX-G is rapidly cleared from the bloodstream, widely distributed across tissues, and capable of crossing the intestinal, blood–brain, and placental barriers [30]. The presence of PnTXs in mollusks could pose a risk to consumers and shellfish farmers, though no human intoxication cases have been reported to date [31]. Moreira-González et al. [32] reported 60 cases of dermatitis in swimmers following exposure to seawater during a *V. rugosum* bloom in Cienfuegos Bay, Cuba.

PnTXs pose a potential public health threat due to their neurotoxic effects, via interaction with nicotinic acetylcholine receptors [33,34]. The study of emerging toxins represents an interesting tool in the discovery of novel potential drugs, in view of their high potency and particular structure.

The aim of this study is to report the discovery of PnTXs and *V. rugosum* in a shellfish farming area in the northwestern Adriatic Sea, during routine monitoring activities.

## 2. Results

Table 2 shows various water and shellfish samples that tested positive for *V.rugosum* and PnTX-G respectively in August 2022. Subsequent samplings, from September to October, revealed no presence of *V. rugosum* in the water column, while traces of PnTx-G were found in clams (Table 2).

### 2.1. Toxin Analyses

Analysis of shellfish samples, specifically Manila clams (*Ruditapes philippinarum*), collected along the Emilia-Romagna coast of Italy, was performed at the Centro Ricerche Marine Laboratory in Cesenatico.

PnTX-G was detected in three samples, with maximum levels observed in August 2022 (6.7 µg/kg for PnTX-G).

**Figure 1:**
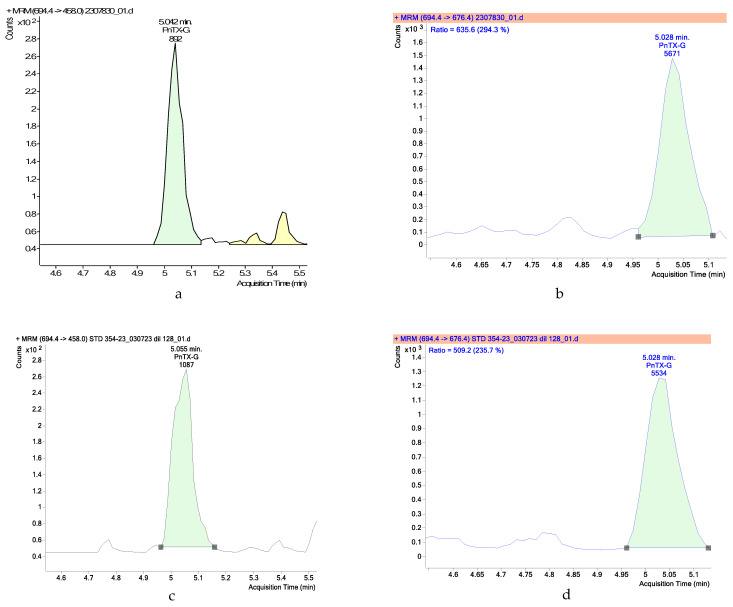
Chromatograms of a sample collected on 22 August 2023. (**a**) PnTX-G: transition 694.4 > 458.0; (**b**) PnTX-G: transition 694.4 > 676.4; (**c**) standard PnTX-G: transition 694.4 > 458.0; (**d**) standard PnTX-G: transition 694.4 > 676.4.

In 2023, a total of five samples of Manila clams were analyzed from June to August. Figure 1 shows the chromatograms of the quantifiable sample for PnTX-G above the limit of quantification (4.5 µg/kg). Another was positive for the presence of PnTX-G but below the limit of quantification.

### 2.2. Microscopy

**Figure 2:**
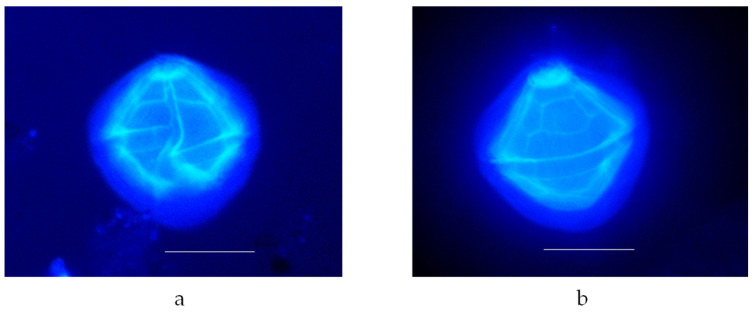
(**a**) Vegetative cell of *V. rugosum* (image captured in fluorescence technique) in ventral view (scale bar: 20 μm); (**b**) vegetative cell of *V. rugosum* (image captured in fluorescence technique) in dorsal view (scale bar: 20 μm).

Optical microscopy analysis, conducted with the epifluorescence technique, shows a characteristic arrangement of thecal plates (Figure 2).

**Figure 3:**
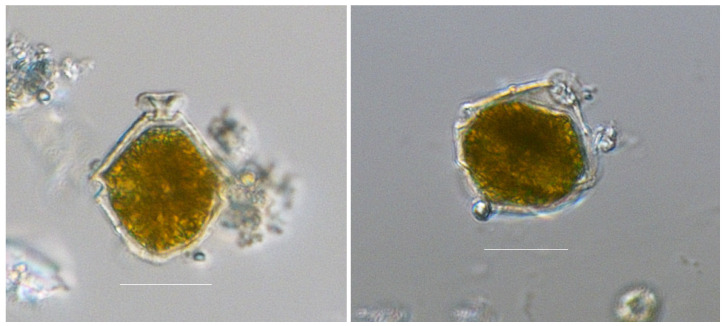
Vegetative cell of *V. rugosum* (fixed with Lugol’s solution) showing a mucoid matrix extruded at the apex (scale bar: 20 μm).

**Figure 4:**
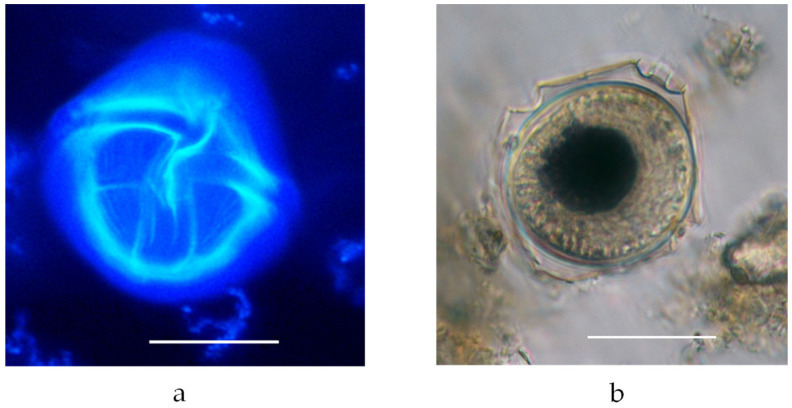
(**a**) Vegetative cell of *V. rugosum* showing the characteristic reticulation of the thecal plates (image in fluorescence technique; ventral view; scale bar: 20 μm); (**b**) cell of *V. rugosum* probably showing transformation in a non-motile stage (cell fixed with Lugol’s solution; scale bar: 20 μm).

*Vulcanodinium rugosum* (Nézan and Chomérat, 2011) is an armored dinoflagellate characterized by a trapezoidal to hemispherical hypotheca and a conical to hemispherical epitheca truncated at the apex (Figure 3). *V. rugosum* exhibits a characteristic large apical pore plate (Po), with a mucous matrix extruded from its center. The thecal surface is covered by longitudinal striae (Figure 4a), often with cross reticulations, and houses numerous golden-brown chloroplasts. The asexual life cycle of *V. rugosum* demonstrated an alternation between a coccoid stage (probably cyst stage) (Figure 4b) and a motile thecate stage [6].

## 3. Discussion

This study reports the first detection of the marine neurotoxin PnTX-G in clams collected in the northwestern Adriatic Sea (Italy). It also represents the first report of the potential toxin-producing dinoflagellate, *Vulcanodinium rugosum*, in Italian seas.

The proposed life cycle for *V. rugosum* [7] involves an alternation of vegetative (motile and pelagic) phases and temporary cysts or non-motile (benthic) cells. To date, there is no specific monitoring for benthic species in bivalve mollusks production areas, and the pelagic stage of *V. rugosum* is difficult to find in the water column. In areas considered potentially at risk (i.e., shallow lagoon areas or otherwise with similar characteristics to those in which the presence of *V. rugosum* has already been described), it may be necessary to implement specific monitoring for benthic species (such as the monitoring already conducted for *Ostreopsis* cf. *ovata* [35]) and, at the same time, monitor PnTXs levels in bivalve mollusks.

The emergence of new biotoxins can be attributed to numerous factors, some of which are not fully understood. Global warming and anthropogenic interventions have facilitated the migration and establishment of emergent toxin producers into Europe’s temperate waters, posing a new threat to public health [10].

The Adriatic Sea is a closed sea that ends in a cul-de-sac to the north, with the only access to the Mediterranean Sea—another closed basin—being through the Strait of Gibraltar to the Atlantic Ocean and through the Suez Canal to the Red Sea in the southern segment (the Strait of Otranto). The introduction of new algal species into this basin is largely due to human activity. With 90% of global commercial transport occurring by sea, the likelihood of exotic aquatic species reaching distant locations from their origin is quite plausible.

As the size of commercial shipping vessels and the global shipping fleet expand, the risk of exotic organisms being introduced into foreign ports via ballast water is on the rise. The ballasting and deballasting of water and residuals by ships in multiple ports are estimated to be the largest contributors to nonindigenous aquatic invasions. In the United States alone, it is estimated that 70 million metric tons of ballast water are discharged into ports annually [36].

The growth of *V. rugosum* is positively correlated with the total concentration of nitrogen, phosphorus, and phosphates. Other factors influencing the development of this dinoflagellate are temperature and salinity [23,24]. Such conditions, which are favorable to the proliferation of *V. rugosum* and have been previously documented in France [23] or in Cuba [32], are also present along the coast of Emilia-Romagna.

The discovery occurred in a private marshland area (fed by seawater) between the provinces of Ferrara and Ravenna, a zone of bivalve mollusk production and, thus, subjected to periodic sampling, in accordance with the surveillance plan of the AUSL (Azienda unità sanitaria locale) of Ferrara.

Since 2022, in this area, in addition to the official monitoring of regulated marine biotoxins, cyclic imines (PnTX-G, gymodimines, spirolides) have also been monitored for research purposes.

From these observations, it can be concluded that both the benthic dinoflagellate *V. rugosum* and the toxins it produces are distributed globally. Specifically, Rambla et al. [37] reported the presence of PnTXs (maximum concentration: 12 µg/kg) in bivalves such as mussel, oyster, and clams from Slovenia, Spain, Ireland, Denmark, Norway, Portugal, and the Netherlands. Hort et al. [38] showed the presence of PnTXs (467.5 µg/kg) in mussels sampled in France (Mediterranean Lagoon), while Norambuena and Mardones [16] found a maximum concentration of 100 µg/kg in mussels from Chile.

It is necessary that a decision be made at the European level to regulate emerging marine biotoxins soon. While the toxic effects of some of these substances on humans are not known, the principle of precaution underpinning legislation, including the so-called Hygiene Package, Regulation (EC) No. 178/2002, should guide our approach. Despite the lack of international regulation for PnTX-G, the French Agency for Food, Environmental and Occupational Health and Safety has suggested that a risk to human consumers may exist if the accumulation of PnTX-G in shellfish exceeds 23 μg/kg [39].

## 4. Materials and Methods

### 4.1. Study Area and Sampling

**Figure 5:**
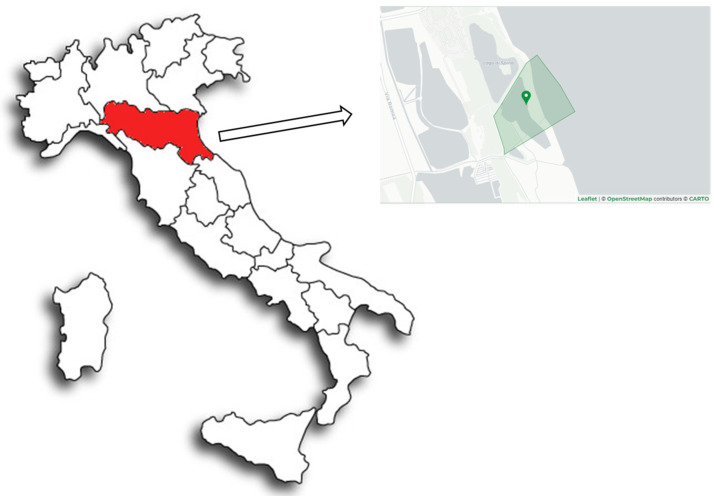
Study area with detail of the basin of drawdown.

The study area is located on the Emilia-Romagna coast, north of the mouth of the Reno River (Lat. N 44°37.798; Long. E 12°14.965), which marks the border between the provinces of Ferrara and Ravenna. It is a natural brackish water basin, fed by sea water through a canal (Gobbino canal) that connects the pond with the sea. The water exchange between the sea and the pond is regulated by a lock. To the south, the pond borders the Bellocchio Veins Nature Reserve (Figure 5).

Seawater samples were collected by a hose sampler to obtain an integrated sample, representative of the entire water column in which the shellfish were growing [40,41].

The harvest of benthic bivalve samples, specifically Manila clams (*Ruditapes philippinarum*) was carried out manually using a rake. For each sample, approximately 30–40 specimens were collected.

### 4.2. Solvent and Reagents

Methanol (MeOH) (LC-MS grade), ammonium acetate, and acetic acid were obtained from VWR (VWR International Srl, Milan, Italy). Water for analysis was supplied by a Millipore system. PnTX-G reference material (CRM) was purchased from NRC Canada (National Research Council, Halifax, NA, Canada).

### 4.3. Toxin Analyses

#### 4.3.1. Extraction

Sample extraction and analysis were carried out according to the harmonized protocol EU-Harmonised-SOP-LIPO-LCMSMS_Version 5 [42].

Chemical analyses were performed at the National Reference Laboratory (NRL) for Marine Biotoxins (Cesenatico, Italy).

The samples were initially rinsed with fresh water and then dissected, before at least 100 g aliquots of mollusk flesh from each sample were homogenized. We added 10 mL of MeOH to an aliquot of shellfish tissue (2 g) and extraction was performed with a high-speed homogenizer (Ultraturrax, IKA, Breisgau, Germany) at 15,000 rpm for 1 min. After the separation of supernatants using a centrifuge (5810R; Eppendorf, Hamburg, Germany) at 4000× *g* for 10 min, extraction was repeated twice, and supernatants were collected in 20 mL volumetric flasks before being made up to 20 mL using MeOH. We filtered 5 µL of the extracts using a 0.2 µm membrane filter (nylon, 13 mm 0.2 µm; Agilent; Santa Clara, CA, USA), which were then were analyzed using LC-MS/MS.

#### 4.3.2. Liquid Chromatography with Tandem Mass Spectrometry (LC-MS/MS)

Analyses of PnTX-G were performed using a UPLC (Infinity II, Agilent, Santa Clara, CA, USA) coupled with a triple-quadrupole mass spectrometer (6460, Agilent, Santa Clara, CA, USA).

Liquid chromatography was performed on a Poroshell120 EC18 (100 × 2.1 mm, 2.7 µm, Agilent, Santa Clara, CA, USA), and separation was achieved using a gradient elution as reported in Table 3, using a mobile phase A (2 mM ammonium acetate, 0.1% *v*/*v* acetic acid in 5.2% *v*/*v* MeOH) and mobile phase B (2 mM ammonium acetate in MeOH).

The flow rate was 0.4 mL/min, the column temperature was 30 °C and samples were refrigerated at 4 °C. The total time was 14 min.

A six-point calibration curve, ranging from 0.4 µg/L to 40 µg/L, obtained by the dilution of CRM with MeOH, was used (*R*^2^ ≥ 0.998) for the quantification of the PnTX-G. The limit of quantification (LOQ) was 4 µg/kg.

The LOQ of 4 µg/kg and the LOD of 1,4 µg/kg were calculated based on the signal to noise ratio (for LOQ, S/N = 10; for LOD, S/N = 3), measured at the minimum concentration level. The identification of PnTX-G was achieved using LC-MS/MS in positive ionization and in the DMRM (dynamic multi reaction monitoring) mode, using the following transitions: 694.4 > 458; 694.4 > 676.4; and 694.4 > 164.1.

The most intense transition, giving the product ion m/z 458, was used to quantify toxin.

The ESI interface was operated using the following parameters: gas temperature, 200 °C; gas flow, 7 mL/min; nebulizer, psi 35; sheath gas heater, 400; sheath gas flow, 12; capillary (V), 4000; vcharing, 300; fragmentor, 225; and three collision energies that were applied (40, 54, and 54 eV).

### 4.4. Identification of Vulcanodinium rugosum

Water samples were concentrated on a nylon filter with a porosity of 20 µm without the use of a pump, so as not to damage any cells that might be present. The filtered material was then transferred (by washing with seawater) to an Utermöhl sedimentation chamber and was analyzed under an inverted light microscope (Nikon Eclipse Ti-U inverted microscope). The samples were inspected in both bright field and phase contrast, using the fluorochrome Fluorescent Brightener 28 (which replaced Calcofluor White), according to the method of Fritz and Triemer [43], for the better visualization of dinoflagellate thecal plates. The fluorochrome employed binds to the polysaccharide components between the plates of armored dinoflagellates. This technique (epifluorescence technique) is used to study the tabulation and structure of the thecate dinoflagellates.

A Nikon Eclipse Ti-U inverted microscope with differential interference contrast was used; the microscope was equipped with a digital camera and epifluorescence system (ultraviolet (UV) 100 W mercury lamp; the UV filter arrangement was 330–380 nm excitation and a 420 nm emission wavelength). The objectives used were 20×, 40×, and 100× (immersion oil).

Taxonomic identification of *V. rugosum* was supported by the previous literature [17,44,45].

## Figures and Tables

**Table 1:** Chemical structure of pinnatoxins.

Toxin	Two-Dimensional Structure
Pinnatoxin-A	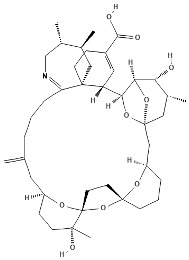
Pinnatoxin-B, -C	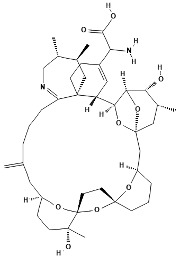
Pinnatoxin-D	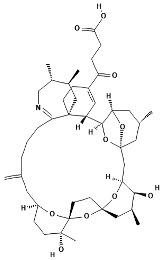
Pinnatoxin-E	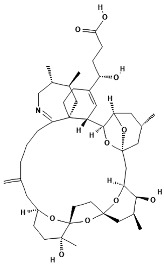
Pinnatoxin-F	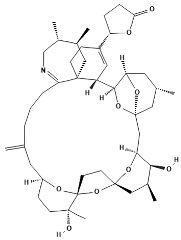
Pinnatoxin-G	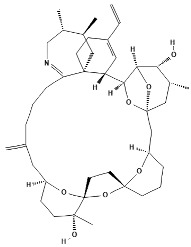
Pinnatoxin-H	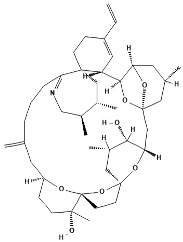

**Table 2:** Determination of PnTX-G concentration in *R. philippinarum* and presence of *V. rugosum* in the collection water.

Sampling Date	PnTX-G (µg/kg SM)	*V. rugosum* (Presence/Absence)
30 August 2022	6.7	Presence
13 September 2022	<LOD	Absence
11 October 2022	<LOD	Absence
21 June 2023	<LOD	Absence
27 June 2023	<LOD	Absence
25 July 2023	<LOD	Absence
22 August 2023	4.5	Absence
29 August 2023	Trace	Absence
04 October 2023	Not collected	Absence
12 December 2023	Not collected	Absence

LOD: limit of detection. SM: shellfish meat.

**Table 3:** Details of the gradient.

Time (Min)	A%	B%	Flow (mL/min)	Max. Pressure (bar)
0	95	5	0.4	1000
1	95	5	0.4	1000
3	37	63	0.4	1000
6	13.8	86.2	0.4	1000
10	13.8	86.2	0.4	1000
10.01	0	100	0.4	1000
11.0	0	100	0.4	1000
11.01	95	5	0.4	1000
14	95	5	0.4	1000

## Data Availability

Data are contained within the article.
